# Building laboratory capacity to support HIV care in Nigeria: Harvard/APIN PEPFAR, 2004–2012

**DOI:** 10.4102/ajlm.v4i1.190

**Published:** 2015-05-14

**Authors:** Donald J. Hamel, Jean-Louis Sankalé, Jay Osi Samuels, Abdoulaye D. Sarr, Beth Chaplin, Eke Ofuche, Seema T. Meloni, Prosper Okonkwo, Phyllis J. Kanki

**Affiliations:** 1 Department of Immunology and Infectious Diseases, Harvard School of Public Health, Boston, MA, United States; 2 Globomics, LLC, Decatur, GA, United States; 3 AIDS Prevention Initiative in Nigeria Ltd. Gte., Abuja, Nigeria; 4 Global AIDS Program Malawi, Centers for Disease Control and Prevention, NICO House Lilongwe 3, Malawi

## Abstract

**Introduction:**

From 2004–2012, the Harvard/AIDS Prevention Initiative in Nigeria, funded through the US President’s Emergency Plan for AIDS Relief programme, scaled up HIV care and treatment services in Nigeria. We describe the methodologies and collaborative processes developed to improve laboratory capacity significantly in a resource-limited setting. These methods were implemented at 35 clinic and laboratory locations.

**Methods:**

Systems were established and modified to optimise numerous laboratory processes. These included strategies for clinic selection and management, equipment and reagent procurement, supply chains, laboratory renovations, equipment maintenance, electronic data management, quality development programmes and trainings.

**Results:**

Over the eight-year programme, laboratories supported 160 000 patients receiving HIV care in Nigeria, delivering over 2.5 million test results, including regular viral load quantitation. External quality assurance systems were established for CD4+ cell count enumeration, blood chemistries and viral load monitoring. Laboratory equipment platforms were improved and standardised and use of point-of-care analysers was expanded. Laboratory training workshops supported laboratories toward increasing staff skills and improving overall quality. Participation in a World Health Organisation-led African laboratory quality improvement system resulted in significant gains in quality measures at five laboratories.

**Conclusions:**

Targeted implementation of laboratory development processes, during simultaneous scale-up of HIV treatment programmes in a resource-limited setting, can elicit meaningful gains in laboratory quality and capacity. Systems to improve the physical laboratory environment, develop laboratory staff, create improvements to reduce costs and increase quality are available for future health and laboratory strengthening programmes. We hope that the strategies employed may inform and encourage the development of other laboratories in resource-limited settings.

## Introduction

Laboratories are fundamental and essential components of health systems, providing clinical staff and patients with test results that are the basis of disease diagnosis and treatment; yet, laboratories are often neglected by governments, development organisations and other stakeholders in plans to improve healthcare systems in developing countries. Despite the scale-up of global health programmes in the last decade, sub-Saharan Africa continues to suffer the consequences of operating with some of the most poorly-equipped and under-resourced laboratories in the world.^1^ As such, by 2012, the US President’s Emergency Plan for AIDS Relief (PEPFAR) Blueprint, the World Health Organization (WHO) and the United Nations Millennium Development Goals (UNMDG) each called for strengthened national laboratory systems as a critical component of scaling up HIV and tuberculosis (TB) prevention and treatment programmes.^2,3^

Based on the Nigerian National HIV Sentinel Surveillance Surveys in 2005 and 2010, the national prevalence of HIV-1 has remained fairly stable at approximately 4%.^4^ The Harvard School of Public Health received PEPFAR funds from 2004 to 2012 to support the development of prevention, care and treatment programmes in Nigeria, Botswana and Tanzania. In Nigeria, Harvard partnered with the AIDS Prevention Initiative in Nigeria (APIN), an organisation developed through funding from the Bill and Melinda Gates Foundation from 2000–2006, to provide evidence-based HIV prevention in four states of the country. The Harvard/APIN PEPFAR programme was built upon this foundation of HIV prevention activities and initiated support of antiretroviral therapy (ART) activities at six tertiary-level facilities in 2004; this expanded to 35 clinics and laboratories by 2009. To ensure sustainability, Harvard helped to establish APIN Ltd./Gte. as an independent, Nigeria-based non-governmental organisation. Beginning in 2009, and fully completed in February 2012, all Harvard/APIN PEPFAR programme activity was transitioned to APIN management.

From the beginning of the Harvard/APIN PEPFAR programme, it was determined that a fundamental component of the capacity-building efforts would be dedicated to laboratory infrastructure, with corresponding growth of logistics management for procuring supplies and laboratory staff training in order to ensure sustainability. In developing the programme frameworks and plans, we incorporated lessons learned from previously-developed ART laboratories in both Nigeria and Senegal so as to elicit lasting gains in laboratory capacity and infrastructure.^5^ In this report, we describe the organisational framework that resulted in the establishment of and continuous quality improvements to laboratory capacity in Nigeria over the eight years of the Harvard/APIN PEPFAR programme (2004–2012). We highlight the collaborative process, with details on specific strategies and methodologies, found to be essential for meaningful laboratory development in a resource-limited setting.

## Research methods and design

**FIGURE 1:**
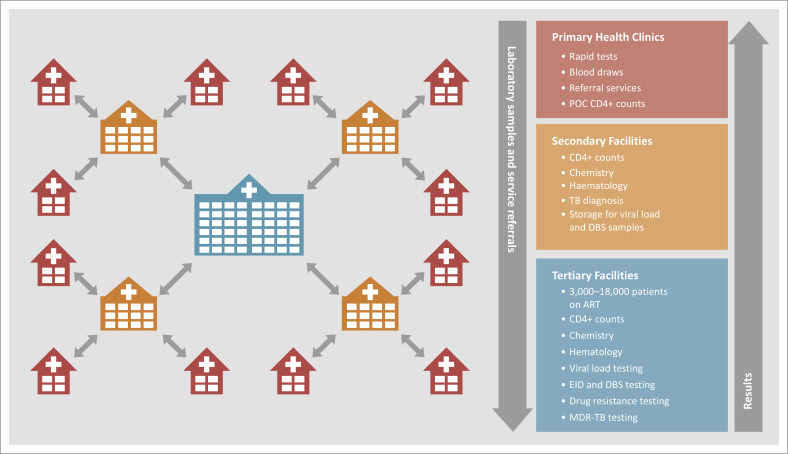
Illustration of hub-and-spoke laboratory organisation.

POC, point-of-care; TB, tuberculosis; DBS, dried blood spots; ART, antiretroviral therapy; EID, early infant diagnosis; MDR, multidrug-resistant.

Our programme’s laboratories were organised with large tertiary facilities at the centre, providing support to secondary hospitals and associated primary health clinics using a hub-and-spoke model (Figure 1). Tertiary-level laboratories were associated with university teaching hospitals or research institutes with large HIV ART programmes. Secondary-level hospitals provided HIV serology, CD4+ cell count enumeration, haematology and clinical chemistry testing. They also had the capacity to store plasma samples for viral load (VL) testing, and dried blood spot (DBS) samples for early infant diagnosis, for up to two weeks before transport to an associated tertiary laboratory. Primary health clinics were smaller health centres that provided basic care, performed HIV rapid testing, drew blood samples for testing to be done elsewhere and referred patients to the secondary or tertiary medical facilities.

### Clinic selection

The selection of a clinic or hospital for development of laboratory capacity to support HIV care was a complex process and required accounting for a number of factors, including patient burden, existing infrastructure, prior collaborations, geographic proximity to other programme facilities and local politics. The Harvard/APIN PEPFAR programme both consulted and collaborated with in-country partners and funding organisations so as to identify candidate clinics. After a clinic was proposed, a detailed site visit was performed to survey the existing laboratory, clinical and personnel infrastructure. There were often substantial obstacles to laboratory development as a result of the poor existing infrastructure, such as the lack of running water or dependable electrical service. Reliability of utilities was essential, as the programme’s protocol for laboratory testing was substantial, requiring electricity-driven instruments (Table 1).

**TABLE 1:** Antiretroviral therapy regimen testing schedule.

Evaluation	0n treatment follow-up schedule†								
	Pre-Entry	Entry‡	3 months	6 months	9 months	12 months	18 months	24 months	Every 6 months
Informed consent	X	X	-	-	-	-	-	-	-
Hepatitis BsAG and Hepatitis C (Ab) testing	-	X	-	-	-	-	-	-	-
Documentation of HIV-1/HIV-2	X	-	-	-	-	-	-	-	-
Medical/medicinal history	X	X	X	X	X	X	X	X	X
Complete physical exam	X	X	-	-	-	-	-	-	-
Targeted physical exam, includes STDs		X	X	X	X	X	X	X	X
Sputum for TB, followed by chest X-ray, referral for TB treatment if needed	X	X	-	-	-	-	-	-	-
Haematology§	X	X	X	X	X	X	X	X	X
Chemistries: ALT, BUN, glucose cholesterol, creatinine††	X	X	-	-	-	-	-	-	-
Follow-up chemistries : ALT, creatinine††, serum/CSF cryptococcal LA test‡‡	-	-	X	X	X	X	X	X	X
Baseline CD4+ cells/mm^3^	X	X							
CD4+ cells/mm^3^	-		X	X	X	X	X	X	X
HIV-1 RNA	-	X	X	X	X	X	X	X	X
HIV-1 resistance genotype (second or more failures only)	-	-	-	X§§	-	-	-	-	-

Ab, antibody; STDs, sexually-transmitted diseases; TB, tuberculosis; ALT, alanine transaminase; BUN, blood urea nitrogen; CSF, cerebrospinal fluid; LA, latex agglutination; CR creatinine ratio.

†, Ideal situation where there is successful virologic and immunologic response to therapy;

‡, Only complete if patient is starting or continuing (government patients) ART;

§, If stable at six months, can omit these and go to every six month schedule;

, May need to be performed outside of schedule if toxicity is suspected;

††, More frequent monitoring of creatinine necessary in patients with CR > 1.5-fold above normal;

‡‡, CSF/serum cryptococcal latex agglutination test only performed when indicated;

§§, Viral genotype performed if: a) viral load > 1000 copies/mL; b) adherent (3 consecutive months’ ART pick-up) and c) is second or more failure.

### Procurement of equipment

Preliminary laboratory improvements began with a needs assessment and included ensuring reliable water and electricity supply, back-up generator, security and adequate air-conditioning capacity. Whilst tertiary and secondary hospital laboratories had an existing patchwork of HIV diagnostics, clinical chemistry, haematology and CD4+ cell count analysers in place, the Harvard/APIN PEPFAR programme expanded and improved access to these critical technologies. In accordance with the WHO’s 2008 Maputo Declaration,^6^ we attempted to provide all laboratories with the same equipment manufacturer and models, supporting standardisation of platforms across sites. Standardisation of laboratory equipment allowed for streamlined training and maintenance, eased acquisition of spare parts and reduced overall costs through higher-volume orders. The availability of in-country servicing along with predicted sustainability of manufacturers, vendors and platforms, were also important factors in selection criteria.

For HIV testing, following the Nigerian national rapid test algorithm guidelines at the time, the Determine HIV rapid test (Alere Medical Co., Japan) was provided, followed by Unigold (Trinity Biotech PLC, Ireland), with Statpack (Chembio Diagnostic Systems, Medford, NY, United States) as the discordant result tiebreaker. If further HIV infection confirmation was required, Western blot (Immunetics, Boston, MA, United States) was performed. Immunologic monitoring of patients’ CD4+ cell counts was performed using the flow cytometry-based Cyflow Counter or Cyflow II (Partec GmbH, Munster, Germany) platform. To monitor virologic treatment response, HIV VLs were measured with the manual COBAS Amplicor HIV-1 Monitor test, version 1.5 (Roche Diagnostics GmbH, Mannheim, Germany). Eligibility for ART and subsequent toxicity were evaluated using relevant blood chemistry assays (Table 1) on the Roche COBAS C311, COBAS C111 or equivalent. Haematology monitoring was performed using the Mindray BC-3200 (Mindray Medical Ltd, Shenzen, China) or equivalent. HIV-1 drug resistance was evaluated, when indicated, using the Viroseq Genotyping System version 2.0 (Abbott Molecular, Des Plaines, IL, United States), with sequencing results being generated on the ABI Genetic Analyser 3130xl (Applied Biosystems, Foster City, CA, United States).

### Laboratory modifications

Many laboratories required physical alterations to existing structures or reconfigurations to improve effective, logical sample processing. A laboratory’s ideal sample flow was established, beginning at the arrival bench, where samples were logged and separated as needed. Sample aliquots were then sent to individual laboratory stations for routine testing, after which samples were moved to storage and to a final station where results were recorded and sent to data entry staff for entry to patients’ records. For more advanced testing, such as deoxyrobinucleic acid polymerase chain reaction (DNA PCR), different steps of the assay protocol were performed in separate rooms, with access restricted to dedicated laboratory members in order to minimise risk of contamination.

Biosafety and fire preparedness procedures were reviewed and revised, and appropriate biohazard waste processing was ensured. Security of laboratories was addressed through both physical and policy improvements, with signage, laboratory renovations and staff trainings that ensured the exclusion of non-essential staff from laboratory spaces. Laboratory data were secured in locked locations with strict access controls and were maintained according to national standards.

### Supply chain

Procurement processes were developed to maximise effective purchasing of equipment and consumables, from expanding use of non-cold chain reagents and regular meetings with in-country laboratory supply sales representatives to working with Supply Chain Management Systems (SCMS) and the Clinton Health Access Initiative (CHAI) so as to secure the necessary test kits. To store all materials for distribution to the sites, two warehouses were maintained – one in the south (Lagos) and one in the centre (Abuja) of the country. A programme logistics manager and head pharmacist were hired and trained, working together to organise and expedite distribution of supplies to the sites using programme vehicles with transport staff and/or by means of an express courier with a negotiated service contract.

### Equipment maintenance

Maintenance of equipment is a critical aspect of ensuring strong laboratory infrastructure, particularly in a resource-limited setting. Most laboratories had dedicated on-site engineers with varying levels of expertise. In addition, programme engineers were hired to travel to other sites for scheduled periodic preventive maintenance as well as specific repairs. Retention of skilled engineers was a serious challenge and concern; accordingly, the programme made great efforts to build local capacity and allow flexible working hours. When soliciting quotes for large equipment purchases for the programme, every effort was made to include training for local engineers and application specialists. Programme engineers also traveled to the United States, Europe and elsewhere in Africa for trainings for specific equipment maintenance on Partec CyFlow analysers (Partec GmbH, Munster, Germany), Nuaire laminar flow hoods (NuAire, Inc., Plymouth, MN, United States) and the Roche COBAS platform (Roche GmbH, Mannheim, Germany).

### Data management

The Harvard/APIN PEPFAR data management team built an easy-to-use, electronic medical records system that allowed for consolidation of laboratory, clinical and pharmacy information using the FileMaker Pro platform (FileMaker Inc., Santa Clara, CA, United States). Wherever possible, these key programme areas were linked by local computer networks within each site. Database plug-ins or utility software tools were designed in order to import electronic laboratory results directly into databases when possible.

Every site also had dedicated data staff to maintain the electronic patient records. All databases were uploaded on a weekly basis to a secure server for compilation by the programme data team, for reporting and monitoring purposes. In addition, all laboratories were equipped with an internet-connected desktop computer for laboratory members to use for programme-related communication and a reference resource.

### Laboratory trainings

The larger tertiary laboratories carried much of the initial responsibility for training and mentoring their smaller secondary and primary satellite laboratories. Programme satellite coordinators were the principal contact persons for all laboratory personnel and communicated problems needing attention to either local site management or up to programme management.

Laboratory quality conferences were held annually in-country, bringing members together from laboratories of every size. Each conference typically had 70 to 90 attendees and were held in various locales within Nigeria. This type of meeting was ideal for advancing overall laboratory quality, addressing changes to programme policy, developing consensus decisions and allowing smaller laboratory groups to interact closely with more experienced peers.

## Results

**FIGURE 2:**
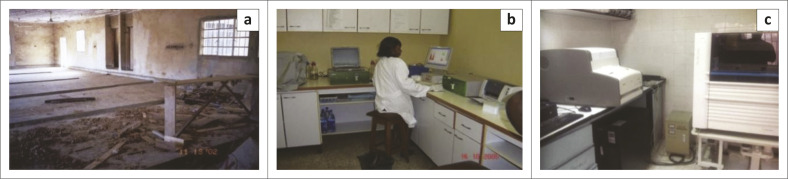
Infrastructure updates made to Jos University Teaching Hospital in Jos, Plateau State.

*Source*: Photo credits: Donald Hamel Laboratory space before (A) and after (B and C) renovations where each space and/or room was designed for specific functions, with deliberate, directional workflows for ideal sample progression.

**FIGURE 3:**
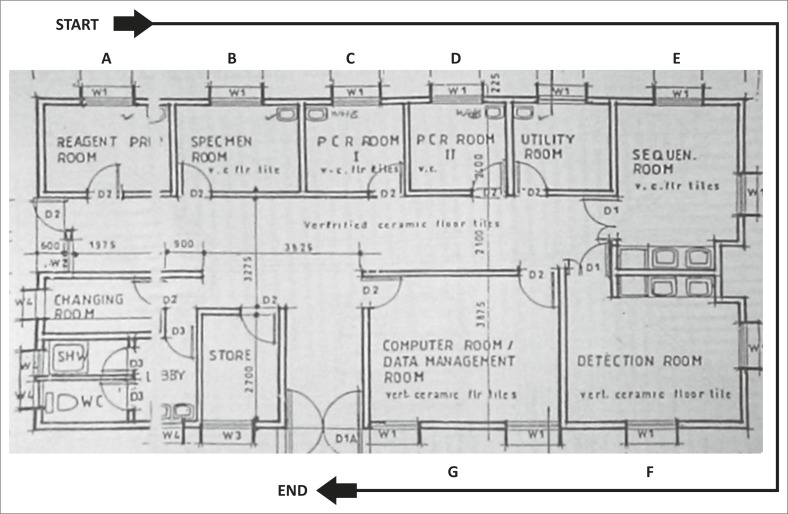
Diagram of the molecular biology laboratory renovations, produced in collaboration with US Centers for Disease Control and Prevention, for directional workflow at the Lagos University Teaching Hospital, Lagos State.

In total, Harvard/APIN PEPFAR helped support and develop the infrastructure at 35 laboratories in Nigeria. Of the 18 major sites managed, 8 were tertiary and 10 were secondary laboratories. In addition, the Nigerian Federal Ministry of Health designated 7 as Centres of Excellence. All laboratories were housed in permanent buildings with electricity, back-up generators, running water and basic infrastructure; a number received substantial upgrades to ensure successful operation and future sustainability. Notable examples of effective laboratory reorganisation were the infrastructure upgrades at the Jos University Teaching Hospital (JUTH) tertiary laboratory (Figure 2) and the logical workflow renovation of the molecular laboratory at the Lagos University Teaching Hospital tertiary laboratory (Figure 3).

A, Clean room for reagent preparation; B, Clean room for performing sample aliquots; C, Clean room for amplification first round of nested polymerase chain reaction (PCR) reactions (clean rooms required a strict policy of no amplified PCR product handling); D, Preparation and amplification room for second round of nested PCR reactions; E, Sequencing reaction room; F, Room for analysis of PCR reactions; G, Workspace for data and image analysis, result reporting and recordkeeping.

All tertiary and secondary laboratory sites provided HIV serodiagnosis through rapid test technologies, automated haematology, clinical chemistry, laser-based CD4+ cell enumeration, VL quantitation and infant DNA PCR diagnosis. The primary laboratories provided access to HIV rapid testing, haematology, clinical chemistry and CD4+ cell count enumeration. Starting in late 2012, APIN upgraded to automated VL equipment, using the COBAS AmpliPrep/TaqMan HIV-1 test, version 2.0. All tertiary and secondary laboratories had the capacity for TB diagnosis with acid-fast bacilli (AFB) staining and a subset of tertiary laboratories developed the infrastructure and capacity for the identification of multidrug-resistant TB (MDR-TB) through various assays, including GeneXpert (Cepheid, Sunnyvale, CA, United States) and Genotype MTBDRplus (HAIN Lifescience GmbH, Nehren, Germany). We introduced screening of select groups at two sites for MDR-TB using the MTBDRplus test and proposed using this test for expanded national surveillance to the National TB Control programme.^7^ Three tertiary laboratories had ABI capillary sequencers (Applied Biosystems, Foster City, CA, United States) for HIV drug resistance testing. In support of the national early infant diagnosis (EID) programme, with the support of CHAI and the US Centers for Disease Control and Prevention (CDC) office in Nigeria, laboratories with PCR testing capacity were also able to provide EID testing of DBS samples. Stock rooms were also improved with greater security mechanisms, such as bars on all doors and windows, sturdy shelving, stock cards and clear ordering and restocking procedures.

By leveraging the high volume of regular laboratory tests required by the programme, contracts were secured for significantly reduced reagent costs from most vendors. By moving from a manual Dynabeads (Life Technologies, Grand Island, NY, United States) method for CD4+ cell count enumeration, to automated Partec CyFlow platforms, test costs were reduced initially from US$22.00/test to US$5.00/test, to a cost in 2012 of under US$2.00/test. The cost of routine chemistry tests, such as alanine aminotransferase (ALT) and creatinine, dropped with the implementation of automated platforms from over US$1.00/test to approximately US$0.29/test. VL test costs using manual Roche Amplicor kits were initially US$33.00/test, but by maintaining a high volume of tests over time and migrating to the automated Roche COBAS platform, costs were reduced to US$14.00/test by 2012.

Starting in 2010, Harvard began shifting laboratory logistics responsibilities to APIN. Existing vendors began to bill APIN directly, and supply chains were modified to increase local procurement of consumables and test kits. Changing import regulations, supplier stock-outs and local strikes necessitated occasional aid from an established non-profit organisation that could provide additional mechanisms for import and customs clearance.

**FIGURE 4:**
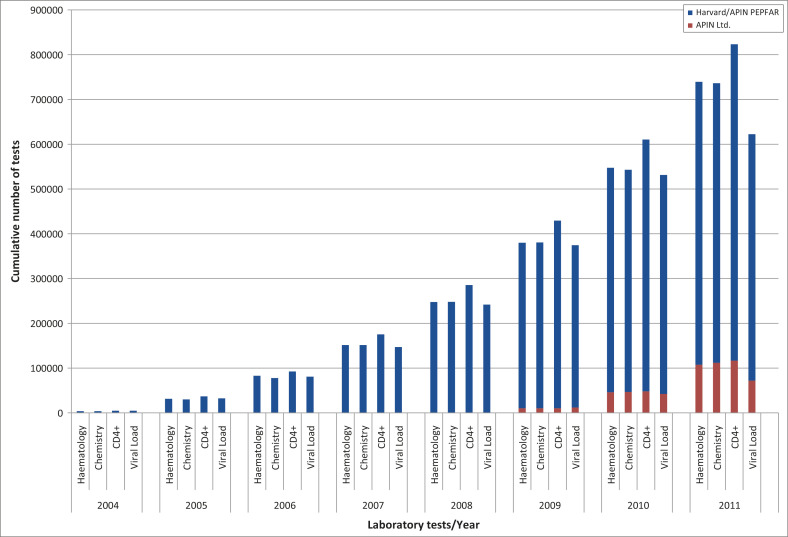
Cumulative laboratory tests performed by Harvard/APIN PEPFAR laboratories.

The achievements of the eight-year laboratory scale-up in the Harvard/APIN PEPFAR programme have been significant. From 2004–2012, Harvard/APIN supported laboratories were able to provide haematology, chemistry, CD4+ cell count enumeration and VL results for over 2.5 million samples (Table 2, Figure 4). The collaboration for EID testing expanded rapidly in Nigeria, with a greater than 10-fold increase in capacity from 2007 to 2008, when over 9000 HIV exposed infants were tested.

**TABLE 2:** Number of tests performed annually by Harvard/APIN PEPFAR laboratories.

Annual Tests	2004	2005	2006	2007	2008	2009	2010	2011
Harvard/APIN PEPFAR							
Haematology	3829	27 595	51 510	68 428	96 324	121 834	131 900	130 743
Chemistry	3622	26 318	47 732	73 688	96 665	122 500	125 643	128 444
CD4+	4905	32 021	55 680	82 765	110 079	133 700	143 204	144 357
Viral Load	4920	27 544	48 543	65 893	94 965	120 765	126 406	61 342
APIN Ltd.							
Haematology	-	-	-	-	-	10 332	35 625	61 417
Chemistry	-	-	-	-	-	10 205	36 709	64 869
CD4+	-	-	-	-	-	10 355	37 913	68 255
Viral Load	-	-	-	-	-	12 007	30 208	29 868

### Impact on health system strengthening

Beyond improvements of specific laboratory services to support the ongoing ART programme, the training and mentorship activities expanded the goal of providing the highest quality of overall healthcare. The programme sought to extend beyond ART delivery and to integrate quality processes and equipment benefits throughout the hospitals and clinics. For example, the purchase of portable TB-diagnostic X-ray equipment became available for use by other hospital departments.

Training of programme staff also benefited the overall institution’s laboratory capacity-building efforts, as most programme staff also had roles in the hospitals’ non-HIV laboratories. Generally, only two to three persons from each laboratory were invited to attend the annual group trainings; subsequently, conference attendees provided step-down training in order to transfer new information to the entire local laboratory team. This also incentivised laboratory managers to maintain high levels of performance, helping to ensure leadership positions for their teams in future trainings. We found the utilisation of training-of-trainer and step-down training methods to be a very cost-effective system of providing training across programme laboratories and allowed for dissemination of training across local laboratory systems.

### Training conferences

Centralised laboratory conferences provided programme management the opportunity to address the laboratory staff teams directly and to acknowledge and commend their hard work. These central workshops allowed programme staff continual assurance of standardisation across programme laboratories. Over time, it was realised that workshop participation could be strengthened through administration of pre- and post-tests encompassing major topics. Additionally, these meetings offered laboratory teams an opportunity to connect with others that were conducting similar work and allowed for generation of a network that could provide local troubleshooting and support. Programme-wide laboratory trainings were attended by 211 laboratory staff over 59 training days; and programme management provided direct laboratory mentorship training over 526 days. In-country teams also developed trainings for more targeted topics such as equipment maintenance, new laboratory platform initiation and accreditation preparedness, training 159 laboratory staff over 84 training days. These numbers do not account for the step-down training days or for retraining sessions that took place upon return to individual laboratory sites.

If programme management identified a laboratory with specific concerns, a site-specific training visit was organised. These localised trainings addressed a wide array of issues, from laboratory staff reorganisation, to launching a trial of a new diagnostic point-of-care platform, to troubleshooting an assay performing out of range. Coordination was sought with clinical and pharmacy training teams so as to extend laboratory topics to their trainings and to apprise laboratory members of any updates to other programme areas that could have an impact on laboratory functions, such as the introduction of new drugs or drug regimens with a specific toxicity concern. Internal and external trainings for laboratory engineers resulted in reduced service calls to factory technicians and less equipment downtime for sites.^8^ Properly-functioning equipment ensured that test kits were consumed in a timely manner, prior to expiration, constituting another cost-saving goal. Additionally, because of both interest and observed need, workshops were held to assist laboratory and research staff with grant writing and publication skill building.

### Electronic data management

Significant gains were achieved in both electronic data capture and improving delivery of laboratory results to clinical staff and patients. Electronic data capture decreased the opportunity of transcription errors and allowed laboratory staff more time for laboratory activities. Laboratory result turnaround times were reduced by an average of two days through the use of electronic data, compared with the prior method that consisted of only handwritten logs and manual transcription.

An advantage of creating a programme-specific database system was that it offered great flexibility, allowing data managers to revise clinical chemistry results to be reported in units that conformed to international standards, or the ability to introduce modifications rapidly as laboratory technologies evolved. For example, the databases were adapted to produce pop-up flags for critical values, such as low haemoglobin or elevated liver or renal enzymes. Another unique electronic tool was the ‘Viral Load Utility’, created to convert analyser data into standardised test results for direct database import. Additionally, the database system was flexible enough to allow for transfer of data to national forms and provide aggregate reporting, when the Federal Ministry of Health developed new registers and aggregate reporting forms.

**FIGURE 5:**
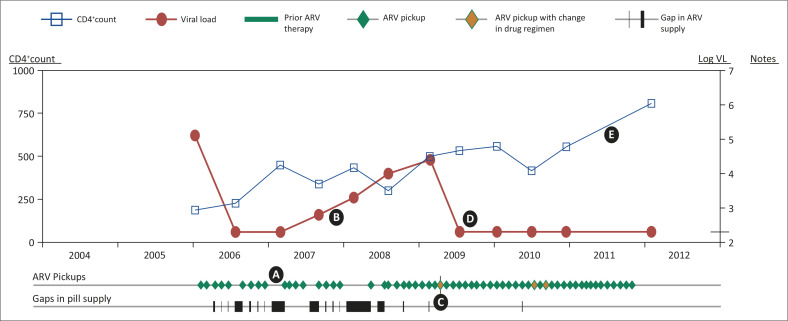
Example of a patient history in the treatment response utility.

Note: Gaps in ARV drug pick up ( A green diamonds) can lead to virologic failure ( B red dots), indicated by increasing viral loads. However, responsive clinical monitoring and a subsequent change in drug regimen ( C yellow diamonds) can return viral supression ( D red dots) indicated by a drop in viral load, and a rise in CD4^+^ counts ( E blue squares)

A major innovation by the Harvard/APIN PEPFAR laboratory and data teams was the design and implementation of an electronic database for both compiling patient laboratory information and then distributing reports over local networks to clinic and pharmacy locations. This ‘Treatment Response Utility’ tool was developed primarily to provide medical staff with a comprehensive picture of a patient’s treatment profile over time and to transfer a greater number of laboratory results presented in a more useful format to the clinical decision-making team (Figure 5).

The utility provides a graphical layout that displays a timeline with VL, CD4+ cell count and drug pick-up data, as well as links to a more detailed clinical history for a particular patient. The visual snapshot aids the physicians in communicating and educating the patient on the positive health effects of ARV drug adherence. The tool also assists clinicians in detecting failure of a drug regimen, assisting in a more rapid switch to a new drug regimen, or intervening for patients struggling with adherence.

### Laboratory quality control and accreditation

At the onset of the Harvard/APIN PEPFAR programme, very few laboratories had quality management systems in place. As laboratories became equipped and staff were trained, data quality processes and laboratory quality assurance systems were instituted. In-country external quality assurance (EQA) was scaled up, with distribution of standardised controls for VL testing. Harvard/APIN PEPFAR-supported laboratories subscribed to EQA programmes distributed by the College of American Pathologists for blood chemistry and viral markers, as well as the United Kingdom National External Quality Assessment Service for CD4+ cell count monitoring. In addition, EQA for EID through DBS testing was conducted through the International Laboratory Branch of the CDC’s Division of Global HIV/AIDS. An EQA programme was not available for the ViroSeq drug resistance genotyping; however, genotyping and sequence analysis were verified at Harvard. Whilst being costly, the international EQA programmes were a requirement of laboratory accreditation programmes, and resulted in marked improvements in reported results for all laboratories by the second year of subscription. Since 2009, addressing long-term sustainability, the Harvard/APIN PEPFAR laboratories have partnered with the Nigerian National External Quality Assessment Laboratory, managed by the Medical Laboratory Science Council of Nigeria, to expand an accredited national EQA programme in lieu of international subscriptions.

The SLMTA programme was launched in Kigali, Rwanda in 2009 by the WHO Regional Office for Africa (WHO AFRO), the CDC, the CHAI and the American Society for Clinical Pathology in an effort to promote accessible pathways to accreditation in sub-Saharan Africa.^1^ Six laboratories from the Harvard/APIN programme were selected for inclusion in the initial SLMTA rollout in 2010. These initial laboratories were nominated by the government of Nigeria and CDC’s office in Nigeria and achieved marked improvements from 2010 to 2012 (Table 3).

**TABLE 3:** SLMTA assessment update of Harvard/APIN PEPFAR-supported labs.

Facility	Baseline stars	Follow-up #1 stars	Follow-up #2 stars	Exit stars	Exit remarks
Ahmadu Bello University Teaching Hospital, Zaria	2	4	4	4	Transitioned to another implementing partner
Jos University Teaching Hospital, Jos	2	4	5	4	Recommended for ISO 15189 accreditation
Lagos University Teaching Hospital, Lagos	2	4	4	4	Sustained improvement
Nigerian Institute of Medical Research (HIV lab), Lagos	2	4	4	4	Sustained improvement
Nigerian Institute of Medical Research (TB lab), Lagos	2	3	3	5	Recommended for ISO 15189 accreditation
University College Hospital, Ibadan	1	4	4	4	Recommended for ISO 15189 accreditation

SLMTA, Strengthening Laboratory Medicine Toward Accreditation; TB, Tuberculosis.

Six tertiary laboratories enrolled in the initial SLMTA rollout in 2010 and achieved exit scores of five stars (one laboratory) and four stars (five laboratories) on a five-star scale. The one five-star laboratory has also been ISO 9001 certified and plans are in place to move additional secondary laboratory sites into future SLMTA quality assessment programmes.^9^ This programme has served as a springboard, not just for the initial laboratories enrolled, but also for all laboratories in the Harvard/APIN programme, to focus on the WHO AFRO assessment scheme and to make dramatic improvements to laboratory quality processes.

## Discussion

Quality laboratory services have become a foundation of the Harvard/APIN PEPFAR programmes in Nigeria and capacity has grown to include automated clinical chemistries and haematology for monitoring ART toxicity at 24 laboratories, PCR-based VL monitoring and EID at 10 laboratories and capillary-based genetic sequencing for HIV drug-resistance mutations at 3 laboratories. Over the course of the programme, the number of patients supported with HIV care rose from 2439 in 2004 to 159 897 by 2012 and our programme laboratories provided over 2.5 million laboratory test results for these patients. In addition, many sites achieved documented improvements in quality services as they moved through the SLMTA programme toward accreditation.

Many of the outcomes detailed in this article originated from major international investments to expand global health programmes in the developing world (eg. PEPFAR). Such scale-ups could represent an opportunity to apply proven methods and novel approaches to effect meaningful improvement in local laboratory capacities. Sustainability of all laboratory improvement endeavours must be considered carefully, with attention being given to the realities of economic constraints, transitions to in-country support and management and integration with national strategic plans. It is critical to build a solid foundation of local laboratory leadership that can maintain improvements independently when international teams depart. The authors believe that without the described improvements to laboratory capacity and quality, the growth and achievements of the Harvard / APIN PEPFAR programme could not have been attained.

Various processes of the 12 quality system essentials that we used to scale up laboratory activities were effective. Specifically, using multiple training methods worked well in ensuring sufficient numbers of trained laboratory staff at each site along with maintenance of high-quality, standardised services throughout the programme sites. The system of centralised procurement and supply distribution allowed for efficient monitoring of supply use and reduction in costs through bulk ordering. By implementing an electronic medical record system, we ensured increased use of data by clinical staff for improved patient care. Furthermore, laboratory teams elevated the overall quality of care at the sites by providing data readily accessed by the electronic Treatment Response Utility. The training efforts also resulted in personnel that were able to develop and maintain laboratories worthy of international accreditation. Additionally, the laboratory scale-up and training efforts had many indirect effects. One major impact of the laboratory scale-up efforts was concomitant health system strengthening across the hospital settings in which our HIV programmes were located. Other groups have documented similarly that the PEPFAR scale-up and integration within existing healthcare systems has improved the linkage of HIV and TB care^10^ and increased the number of in-hospital births in resource-limited settings.^11^

In addition, as a result of PEPFAR-associated training efforts, various programme-affiliated laboratory researchers from Nigeria have been successful in gaining Fogarty Fellowships (Fogarty International Center, NIH, Bethesda, MD, United States) to spend several months working on research projects in the Harvard research laboratories in Boston, MA, United States. The Harvard/APIN PEPFAR team has published research in peer-reviewed journals supporting the cost effectiveness and patient benefit from regular VL and drug resistance monitoring.^12,13^ Finally, Harvard worked with APIN to expand testing capabilities by distribution of point-of-care equipment (including the Partec CyFlow miniPOC) and are embarking on a new programme to test a point-of-care technology for measuring VLs in an African resource limited setting.^14^

## Conclusion

In this article we have provided an overview of methods that may be useful in the development and support of a sustainable laboratory infrastructure, whilst simultaneously developing quality processes through a quality management system model and building upon the existing physical and human capital in a resource-limited setting such as Nigeria. Looking forward, as PEPFAR’s reorganisation of management by implementing partners occurs, APIN endeavours to apply these strategies to strengthen laboratories at the hospitals it inherits and hopes that new partners at ceded locations continue to do the same. We hope that many of these lessons learned and strategies employed may assist and encourage the development of other laboratories in resource-limited settings.
